# Distinguishing Between Bacterial and Viral Community‐Acquired Pneumonia in Hospitalized Adults: The Importance of Clinical, Laboratory, and Imaging Findings

**DOI:** 10.1155/pm/8552717

**Published:** 2026-06-21

**Authors:** Daria Strelkova, Anastasia Yasneva, Olga Kupriushina, Sergey Avdeev, Anna Vlasenko, Lyudmila Fedina, Olga Ivanova, Ivan Zakharenkov, Inna Kaledina, Nataliia Ananicheva, Svetlana Rachina

**Affiliations:** ^1^ Department of Hospital Therapy #2, I.M. Sechenov First Moscow State Medical University, Moscow, Russia, mma.ru; ^2^ Pulmonology Department, I.M. Sechenov First Moscow State Medical University, Moscow, Russia, mma.ru; ^3^ Center for Evidence-Based Medicine and Biostatistics, Samara State Medical University, Samara, Russia, samsmu.ru; ^4^ Clinical Pharmacology Department, Clinical Hospital named after S. S. Yudin, Moscow, Russia; ^5^ Pulmonology Department, Branch No. 4 of the Federal State Institution “1586 Military Clinical Hospital” of the Ministry of Defense of the Russian Federation, Smolensk, Russia; ^6^ Intensive Care Unit #9, City Clinical Hospital named after S. S. Yudin, Moscow, Russia; ^7^ Intensive Care Unit #7, City Clinical Hospital named after S. S. Yudin, Moscow, Russia; ^8^ Regional Vascular Center, City Clinical Hospital named after S. S. Yudin, Moscow, Russia

## Abstract

**Background:**

Our study aims to compare patients with identified viral and bacterial pathogens and develop a predictive model for determining community‐acquired pneumonia (CAP) etiology.

**Methods:**

In this retrospective case–control study, hospitalized adults with confirmed bacterial or viral CAP were compared at a 1:3 ratio. Patients were matched by gender, hospitalization department, age, and Charlson comorbidity index. The etiology of CAP was established through blood and sputum culture, polymerase chain reaction of respiratory specimens, and urinary antigen tests. Both objective variables and subjective symptoms were assessed using multivariate conditional logistic regression.

**Results:**

Our study included 400 patients. Using the best‐developed model, we found that patients with bacterial CAP were more likely to exhibit unilateral infiltration on visualization (odds ratio [OR] 20.47; 95% confidence intervals [CI; 7.51–55.81], *p* < 0.001), experience chills (OR 20.69; 95% CI [6.08–70.38], *p* < 0.001), have a higher heart rate (OR 1.04; 95% CI [1.01–1.06], *p* = 0.001), and demonstrate an elevated neutrophils/lymphocytes ratio (OR 1.58; 95% CI [1.16–2.14], *p* = 0.003). They also showed a decreased level of consciousness (OR 3.16; [1.16–8.61], *p* = 0.024), and were more likely to receive vasopressors within the first 24 h of admission (OR 8.28; 95% CI [1.75–39.22], *p* = 0.008). Conversely, they were less likely to have general weakness (OR 0.20; 95% CI [0.09–0.47], *p* < 0.001) and tended to have lower total serum protein levels (OR 0.06; 95% CI [0.01–0.45], *p* = 0.006). This model showed sensitivity of 90%, specificity of 91%, and positive predictive value of 97%.

**Conclusion:**

The developed model offers a valuable tool for assessing the etiology of CAP upon admission.

## 1. Introduction

Community‐acquired pneumonia (CAP) stands as one of the most prevalent infectious diseases, imposing a tremendous socioeconomic burden [1, 2]. The advent of the coronavirus disease 2019 (COVID‐19) pandemic has underscored the problem of discerning between lung injuries of various etiologies in adults, necessitating different care conditions and antimicrobial therapy approaches.

During the COVID‐19 pandemic, the widespread use of antibiotics in patients with viral pneumonia has increased greatly. According to meta‐analyses, antibiotics were administered in 74.6%–89.0% of hospitalized patients with COVID‐19, with these numbers being higher in lower and middle‐income countries [3, 4]. In Russia, the overall antibiotic consumption increased during COVID‐19 pandemic [5–7]. This has contributed to further spread of antibiotic resistance.

Numerous studies have endeavored to compare clinical signs and symptoms [8–11], laboratory inflammatory markers [12–15], and imaging findings [16–19] among patients with viral and bacterial pneumonia. However, very few of them, mostly in children, have delved into the efficacy of amalgamating various diagnostic tools (clinical, laboratory, and instrumental) to differentiate between bacterial and viral etiologies of CAP [20, 21].

This study aimed to identify the most promising yet routinely available features for differentiating between bacterial and viral CAP in adult inpatients and to construct a predictive model for its bacterial etiology.

## 2. Methods

### 2.1. Study Design

In a retrospective case–control study, we enrolled hospitalized adults aged ≥ 18 years with confirmed diagnosis of bacterial or viral CAP in a 1:3 ratio. Patients were driven from databases of multidisciplinary hospitals in Moscow (two centers), Krasnodar (one center), Novosibirsk (one center), Murmansk (one center), Smolensk (three centers), Yakutsk (one center), and Obninsk (one center) spanning the period from 2014 to 2023. Gender, hospitalization department, age, and Charlson comorbidity index [22] were used for patient matching.

### 2.2. Inclusion/Exclusion Criteria

**Figure 1:**
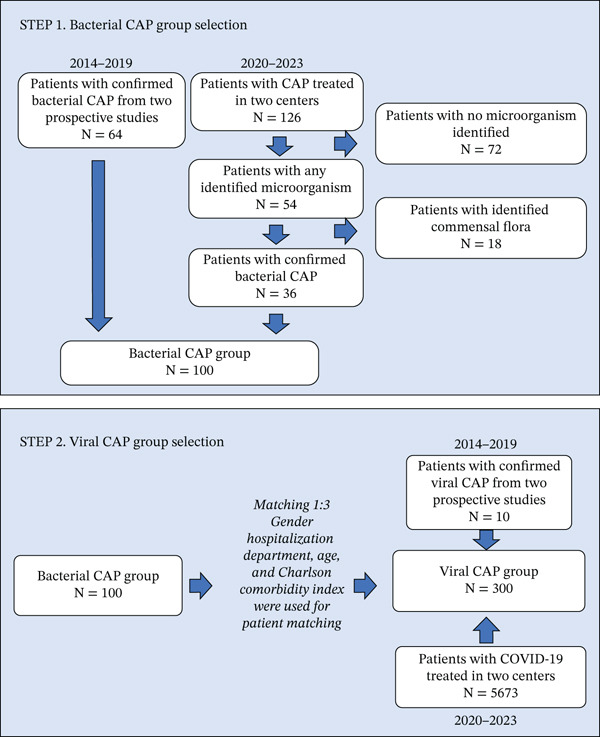
Patients’ selection process. Step 1: The bacterial CAP group included patients with confirmed bacterial CAP from prospective studies of CAP etiology (2014–2019) and retrospective analysis of 2020–2023 CAP cases from two study centers. Step 2: The viral CAP group was obtained by 1:3 matching to the bacterial CAP group based on gender, hospitalization department, age, and Charlson comorbidity index, including patients with confirmed viral CAP (2014–2019) and retrospective analysis of 2020–2023 COVID‐19 cases from two study centers.

The diagnosis of both bacterial and viral CAP relied on conventional criteria (infiltrates observed on chest radiography or computed tomography (CT), along with signs and symptoms consistent with lung involvement and/or laboratory abnormalities indicative of the systemic inflammatory response) in accordance with national guidelines [23]. The patients’ inclusion process is shown in Figure 1.

The etiology of CAP was determined through several methods, including blood culture, gram stain, and culture of respiratory specimens (such as sputum, endotracheal aspirate, or bronchoalveolar lavage), as well as *Streptococcus pneumoniae* and *Legionella pneumophila* serogroup 1 urinary antigen tests. Real‐time polymerase chain reaction was applied for lower respiratory tract samples or combined naso‐/oropharyngeal swabs to identify deoxyribonucleic acid/ribonucleic acid of *Mycoplasma pneumoniae*, *Chlamydia pneumoniae*, *L. pneumophila*, and various respiratory viruses, including influenza viruses A and B, human respiratory syncytial virus (RSV), human adenovirus, human metapneumovirus, human coronaviruses (229E, HKUI, OC43, NL63), human parainfluenza virus‐1‐4, human rhinovirus, human bocavirus, and severe acute respiratory syndrome‐related coronavirus 2 (SARS‐CoV‐2), since 2020. Only cases with a definite and probable etiology of CAP were considered suitable for inclusion. Patients with identified commensal flora were excluded.

### 2.3. Data Collection

The medical records of the patients were meticulously reviewed. For each patient’s medical history, vital signs, physical examination findings, demographic and laboratory data, chest radiography, and/or CT obtained within the first 24 h, in‐patient pharmacological and non‐pharmacological treatment, and outcome of hospital stay, were documented and analyzed.

### 2.4. Statistical Analysis

Continuous data were shown as mean ± standard deviation (Me (SD)) or median with interquartile range (Med (Q1; Q3)), and categorical data were presented as frequencies and percentages. Normality was assessed using the Shapiro–Wilk test. Differences between patients with viral and bacterial CAP were analyzed using conditional logistic regression, with stratification by sex, age, comorbidity index, and hospital department. The conditional logistic regression approach was selected to account for potential confounding by demographic and clinical characteristics, providing more robust estimates than separate univariate tests. All differences were considered statistically significant when the *p* value was less than the predetermined level of significance (0.05) in two‐tailed tests. Balance between groups was further assessed by calculating the standardized mean difference (SMD).

The modeling method chosen was multivariate conditional logistic regression with backward stepwise selection. Objective variables (such as unilateral infiltration on chest X‐ray, neutrophil/lymphocyte ratio, etc.) and a combination of objective and subjective variables, including patients’ complaints (such as chills, general weakness, etc.) were separately considered during the model development. The logistic regression equation is presented below, where *P* represents the probability that pneumonia has bacterial etiology and *Z* is the regression coefficient reflecting the degree of influence of all input factors:

P=11+e−Z,

The classification quality was assessed using cross‐validation on five subsample folds. The whole initial data set was divided into five parts, with model building performed on four of them, while the 5th part was used for validation. This process was repeated until validation was performed on all five subsamples. The evaluated quality metrics included the area under the receiver‐operating characteristic (ROC) curve (AUC), the area under the precision‐recall curve (AUC‐PR), classification accuracy (CA), sensitivity, specificity, and positive predictive value (PPV). The ROC curves are presented in accordance with the TRIPOD guidelines [24]. Calculations and graphics were performed in R using the survival (v.3.5.7) package for conditional logistic regression and caret (v.6.0.94) for cross‐validation procedures. Two methods of handling missing data were employed: removal of individuals with missing data and median imputation.

### 2.5. Ethical Statement

The present study protocol was reviewed and approved by the Local Ethics Committee of I.M. Sechenov First Moscow State Medical University, Moscow, Russian Federation (Ethics Approval Letter № 11‐23; dated: 15.06.2023).

## 3. Results

A total of 400 patients were enrolled in the study. Baseline characteristics of patients in both groups are presented in Table 1. Bacterial CAP presented more severely, with higher rates of dyspnea, sputum production, pleuritic chest pain, chills, impaired consciousness, and worse vital signs, whereas viral CAP more often manifested with general weakness and upper respiratory symptoms (anosmia, rhinitis). Imaging differed markedly: viral CAP was more often associated with bilateral infiltrates (89% vs. 51%) and ground‐glass opacities (95% vs. 34%), while bacterial CAP showed more unilateral involvement, consolidations (72% vs. 45%), and pleural effusion (26% vs. 10%). Inflammatory markers were also higher in bacterial CAP, including leukocyte count (10.0 (5.6; 15.1) vs. 6.2 (4.7; 9.0) × 10^9^/L), CRP (129.9 (33.9; 284.4) vs. 78.4 (35.4; 147.3) mg/L), and procalcitonin elevation (74% vs. 17%).

**Table 1:** Patients’ main characteristics.

	Bacterial CAP (*n* = 100)	Viral CAP (*n* = 300)	*p* value	*SMD*
Demographic data				
Age, years	51.0 (37.8; 67.0)	51.5 (39.0; 67.2)	—	−0.07
Male, *n* (%)	53 (53%)	143 (47.7%)	—	0.09
Admission to ICU	66 (66%)	201 (67%)	—	0.02

Main comorbidities				
Charlson comorbidity index, points	2.0 (0.0; 4.0)	2.0 (0.0; 4.0)	—	0.03
Arterial hypertension, *n* (%)	27 (27%)	186 (62%)	< 0.001	−0.74
Congestive heart failure, *n* (%)	18 (18%)	27 (9%)	0.03	0.25
Chronic obstructive pulmonary disease, *n* (%)	16 (16%)	15 (5%)	< 0.001	0.37
Chronic hepatitis, *n* (%)	15 (15%)	6 (2%)	< 0.001	0.47
Diabetes mellitus, *n* (%)	13 (13%)	51 (17%)	0.30	−0.12
Stroke or transient ischemic attack, *n* (%)	11 (11%)	39 (13%)	0.56	−0.06
Chronic kidney disease, stage С3а‐С5, *n* (%)	9 (9%)	36 (12%)	0.42	−0.09
Coronary artery disease, *n* (%)	9 (9%)	33 (11%)	0.51	−0.06

Outcome				
Hospital mortality, *n* (%)	30 (30%)	130 (43%)	0.01	−0.27
Transfer from general ward to ICU, *n* (%)	4 (4%)	40 (13%)	0.01	−0.33

The most common bacterial pathogen was *S. pneumoniae*, followed by *Klebsiella pneumoniae* and *Staphylococcus aureus*. Mixed bacterial etiology was observed in 23 (23%) cases (see Table S2). SARS‐CoV‐2 was predominant in the viral CAP group, with 290 (96.6%) patients affected. In the remaining cases, the identified pathogens included rhinovirus (three cases), metapneumovirus (two cases), influenza A virus (two cases); as well as RSV, other human coronaviruses, influenza B virus + RSV, each accounting for one case.

There were no statistically significant differences in age, gender, unit of admission (ICU vs. general ward), or Charlson comorbidity index between the two groups. Hospital mortality rates were 30% in bacterial CAP and 43%—in viral CAP, respectively. A detailed description and comparison of the two cohorts of patients is provided in Table S1.

Results of the univariate conditional logistic regression analysis are shown in Table 2. Concomitant diseases such as chronic obstructive pulmonary disease and congestive heart failure, chills, pleuritic chest pain, runny nose, presence of sputum, and decreased level of consciousness were found to be more common in bacterial CAP, whereas general weakness and anosmia were more characteristic of viral CAP. Unilateral infiltration, presence of pleural effusion, and a higher heart rate on ECG were likely to be associated with bacterial rather than viral CAP.

**Table 2:** Univariate logistic regression analysis of probability of bacterial vs. viral CAP in adults.

Variable	Odds ratio [95% CI]	*p* value
Comorbidities		
Chronic obstructive pulmonary disease	3.82[1.78–8.21]	< 0.001
Congestive heart failure	2.09[1.10–3.99]	0.025

Scales		
CURB 65	2.71[2.00–3.67]	< 0.001

Signs and symptoms		
Chills	29.71[11.98–73.68]	< 0.001
Pleuritic chest pain	11.75[6.16–22.43]	< 0.001
Runny nose	7.77[2.90–20.79]	< 0.001
Presence of sputum	3.72[2.28–6.06]	< 0.001
General weakness	0.08[0.04–0.15]	< 0.001
Anosmia	0.08[0.001–0.60]	0.007
Decreased level of consciousness	5.95[2.87–12.34]	< 0.001

Instrumental investigations		
Unilateral (chest X‐ray/CT) infiltration vs bilateral	25.48[12.13–53.56]	< 0.001
Pleural effusion	2.95[1.64–5.33]	< 0.001
Heart rate on ECG	1.05[1.03–1.06]	< 0.001

Laboratory findings		
Blood leukocytes	1.50[1.16–1.96]	0.002
Blood neutrophils	1.42[1.09–1.85]	0.009
Blood neutrophils/lymphocytes ratio	1.56[1.27–1.93]	< 0.001
Serum CRP	1.30[1.07–1.58]	0.008
Serum creatinine	2.02[1.48–2.75]	< 0.001
Serum urea	2.19[1.68–2.87]	< 0.001
Total serum protein	0.03[0.01–0.12]	< 0.001
Proteinuria	0.47[0.28–0.77]	0.003
Leukocyturia	0.45[0.21–0.96]	0.038
Ketonuria	0.46[0.23–0.94]	0.033

Treatment		
Mechanical ventilation	4.36[2.20–8.63]	< 0.001
Vasopressors in 24 h of admission	11.57[3.64–36.77]	< 0.001

*Note:* For each quantitative variable OR was calculated for its 2‐fold increase.

Abbreviation: CI – confidence intervals.

The bacterial CAP group exhibited higher levels of leukocytes, neutrophils, serum C‐reactive protein (CRP), creatinine, and urea, along with lower levels of total protein. Urine sediment abnormalities were more frequently detected in viral CAP cases. Patients with bacterial CAP were also more likely to require vasopressors and mechanical ventilation upon admission.

The results of multivariate conditional logistic regression are displayed in Table 3. Overall, unilateral infiltration on chest radiography/CT, vasopressor use in the first 24 h of admission, higher heart rate, and chills emerged as the most significant factors in distinguishing bacterial and viral CAP.

**Table 3:** Multivariate conditional logistic regression analysis of probability of bacterial vs. viral CAP in adults.

Variable	Removed missing data		Median imputation	
	Odds ratio [95% CI]	*p* value	Odds ratio [95% CI]	*p* value
Objective variables				
Unilateral infiltration (chest X‐ray/CT)	73.70[19.16–283.40]	< 0.001	32.14[12.22–84.52]	< 0.001
Vasopressors in 24 h of admission	58.13[5.18–652.60]	0.001	13.82 [3.28–58.29]	< 0.001
Heart rate on ECG	1.05 [1.03–1.08]	< 0.001	1.04 [1.02–1.06]	0.001
Decreased level of consciousness	4.32[1.28–14.61]	0.006	3.56 [1.36–9.29]	0.010
Blood neutrophils/lymphocytes ratio	1.32[1.13–2.33]	0.002	1.62 [1.22–2.16]	0.001
Total serum protein level	0.03[0.001–0.35]	0.016	0.03 [0.00–0.18]	< 0.001
Serum urea level	—	—	1.81 [1.25–2.60]	0.002
Proteinuria	—	—	0.31 [0.14–0.67]	0.003

Objective variables and subjective symptoms				
Vasopressors in 24 h of admission	45.03[2.18–930.77]	0.014	8.28 [1.75–39.22]	0.008
Unilateral infiltration (chest X‐ray/CT)	80.62[15.74–412.99]	< 0.001	20.47 [7.51–55.81]	< 0.001
Chills	15.89[1.89–133.64]	0.011	20.69 [6.08–70.38]	< 0.001
Heart rate on ECG	1.05 [1.02–1.08]	0.001	1.04 [1.01–1.06]	0.001
Decreased level of consciousness	3.35 [0.95–11.86]	0.051	3.16 [1.16–8.61]	0.024
Blood neutrophils/lymphocytes ratio	1.82 [1.18–2.81]	0.007	1.58 [1.16–2.14]	0.003
General weakness	0.14 [0.04–0.45]	0.001	0.20 [0.09–0.47]	<0.001
Total serum protein level	0.10 [0.01–1.07]	0.053	0.06 [0.01–0.45]	0.006

The logistic regression equations for objective variables, with missing data removed and replaced by median values, are presented in Formulas 1 and 2 in the Supporting Information.

**Figure 2:**
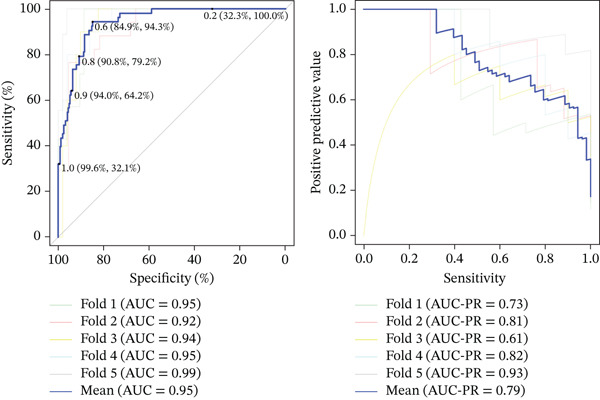
The AUC and AUC‐PR for model predicting bacterial vs. viral CAP based on objective variables (dataset with removed missing data).

**Figure 3:**
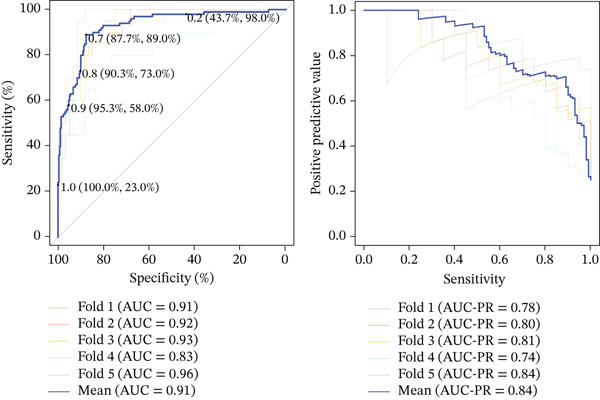
The AUC and AUC‐PR for model predicting bacterial vs. viral CAP based on objective variables (dataset with replacement of missing data with the median values).

The AUC and the AUC‐PR for each fold, as well as the average of all folds are depicted in Figure 2 (for the dataset with missing data removed) and Figure 3 (for replacement of missing data with median values), respectively.

When missing data were removed, high AUC values were observed, ranging from 0.92 to 0.99, with a mean of 0.95. However, moderate AUC_PR values ranging from 0.61 to 0.93 were observed, with a mean of 0.79. Overall, the CA was 86%, sensitivity was 94%, specificity was 85%, and PPV was 98% (using a cut‐off point of 0.60). In the case where missing data were replaced by median values, there was a decrease in AUC from 0.95 to 0.91, along with a concomitant increase in AUC‐PR, from 0.79 to 0.84. The CA increased to 88%, sensitivity decreased to 89%, specificity increased to 88%, and PPV was 96% (using a cut‐off point of 0.67).

The values of the coefficients of the logistic regression equation for the combination of objective and subjective variables are presented in Formulas 3 and 4, Supporting Information.

**Figure 4:**
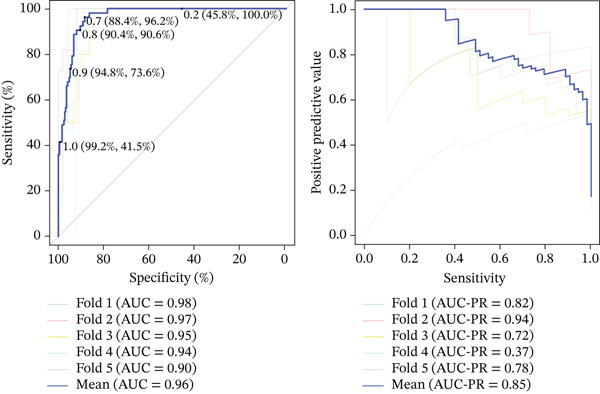
The AUC and AUC‐PR for the model predicting bacterial vs. viral CAP based on objective and subjective variables (dataset with removed missing data).

**Figure 5:**
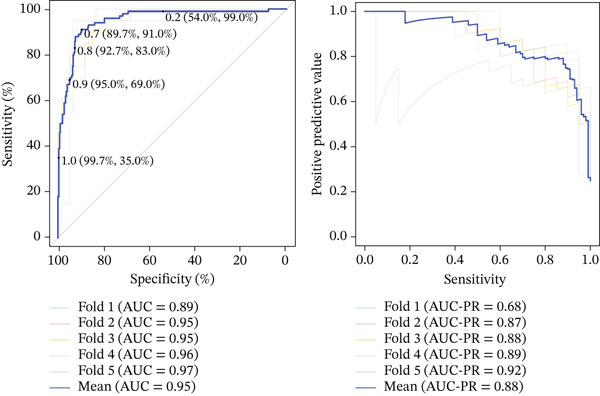
The AUC and AUC‐PR for the model predicting bacterial vs. viral CAP based on objective and subjective variables (dataset with replacement of missing data with the median values).

The AUC and AUC‐PR for each fold, as well as the average of all folds, are shown in Figure 4 (for the dataset with missing data removed) and Figure 5 (for replacement of missing data with median values), respectively.

After incorporating subjective variables into the model and excluding patients with missing data from the analysis the model showed the following parameters: AUC = 0.96 (0.94 to 0.98), AUC_PR = 0.85 (0.37 to 0.94), CA—90%, sensitivity—88%, specificity—96%, PPV—99% (using a cut‐off point of 0.72). When combining objective findings, subjective symptoms, and the method of substituting missing values with median, the model quality was as follows: AUC = 0.95 (0.89 to 0.97), AUC_PR = 0.88 (0.68 to 0.92), CA—90%, sensitivity—90%, specificity—91%, and PPV—97% (using a cut‐off point of 0.68).

Nomograms developed for the convenient application of these models are presented in Figures S1–S4.

## 4. Discussion

In our study, we conducted a comparison between two well‐defined cohorts of inpatients with confirmed etiology of CAP, aiming to identify the most characteristic clinical, laboratory, and instrumental parameters and develop a model for distinguishing between bacterial and viral CAP.

The matching of these two groups revealed several consistent correlations with the causative agent of pneumonia. Factors such as the extent of lung infiltration on сhest X‐ray/CT (unilateral vs. bilateral), total serum protein, neutrophil/lymphocyte ratio, level of consciousness, heart rate, and vasopressor use on the first day of hospital admission consistently demonstrated significant associations with the etiology of CAP. Importantly, these associations remained statistically significant regardless of the input data set and method used to handle missing data. It should be noted that serum urea level and the presence of proteinuria exhibited correlations with the causative agent of CAP, albeit with certain limitations, particularly when missing data were replaced my medians and only objective variables were considered. This suggests that the identified relationship may be unstable and requires additional proof in future studies and/or external validation.

As mentioned, key clinical features distinguishing bacterial CAP from viral included the presence of chills, decreased level of consciousness, and the absence of general weakness. These findings align with previous research, where other investigators have also noted the prominence of these symptoms in bacterial CAP compared with COVID‐19 [9, 11]. In addition, some studies have characterized bacterial pneumonia by the presence of productive cough and chest pain [8–11].

It is noteworthy that adding patient complaints into the model, such as chills and general weakness, slightly improved its diagnostic performance. Bhuiyan M.U. et al. obtained similar results in their study, where combining elevated CRP with either the presence of fever (≥ 380°C) or the absence of rhinorrhea improved the differentiation between bacterial and presumed viral pneumonia compared with CRP alone [21]. However, it is important to acknowledge that their study was performed in a pediatric population, limiting direct comparisons.

In the meantime, several tools have been developed for adults to assess the likelihood of *M. pneumoniae* pneumonia, which involve the simultaneous evaluation of patient complaints along with additional instrumental and/or laboratory abnormalities [25, 26]. Ren Y. et al. proposed a nomogram prediction model based on age, body temperature, dry cough, dizziness, CRP level, and the “tree‐in‐bud” radiological sign, which demonstrated high accuracy in diagnosing CAP, associated with *M. pneumoniae* [26].

The radiological changes characteristic of different etiologies of CAP in our study were generally consistent with previously published data. In bacterial pneumonia, features such as unilateral infiltration, pleural effusion, and consolidations as well as the absence of ground glass opacity were noted more frequently compared with COVID‐19 [11, 14, 17, 27]. Notably, the most significant discriminatory feature between bacterial and viral CAP included in our model was the unilateral pattern of lesions.

It should be noted that previous attempts to use lung CT to predict the etiology of CAP in adults were made before the emergence of COVID‐19. For instance, Ono A. et al. compared chest CT findings in adults with seasonal influenza and pneumococcal CAP [18]. They found that patients with influenza virus CAP were more likely to exhibit ground‐glass attenuation and crazy‐paving appearance, whereas consolidations, bronchial mucus plugging, centrilobular nodules, and pleural effusion were more common in cases of S*. pneumoniae* pneumonia.

There is a possibility that the prospects of using CT to differentiate CAP of various etiologies will be associated with the introduction of artificial intelligence and neural networks. Based on available data, such strategies demonstrate high sensitivity and specificity for the diagnosis of COVID‐19 versus bacterial CAP [19, 28, 29].

Among the laboratory parameters included in the developed models, total serum protein and the neutrophil/lymphocyte ratio showed a stable relationship for association with CAP etiology. However, urea level and proteinuria exhibited the least stable relationship. Notably, the observed trends towards a change in urine analysis (presence of proteinuria) in patients with viral CAP have not, to our knowledge, been previously described in the literature.

The potential use of leukocytes, neutrophils, lymphocytes, and monocytes in the differential diagnosis of bacterial and viral CAP has been described by many researchers [10, 11, 13, 30]. Wang J. et al. also observed a propensity for lower total serum protein levels in patients with bacterial and conventional viral CAP compared with COVID‐19. The differential diagnosis model developed by this group of researchers included eosinophils, total protein, prealbumin, potassium, high‐density lipoprotein cholesterol, and low‐density lipoprotein cholesterol levels and was shown to be highly accurate at identifying COVID‐19 pneumonia [31].

Although in many studies, including ours, CRP levels were significantly higher in patients with bacterial vs. viral CAP [11, 13, 32], determining the optimal distinguishing cut‐off value remains challenge choosing. Similar difficulties in differentiation bacterial CAP from viral lung lesions have been encountered for procalcitonin [15, 33].

Simultaneous determination of ferritin and procalcitonin may be more reliable. For instance, Gharamti A.A. et al. found that a ferritin/procalcitonin ratio ≥877 detected COVID‐19 infection with a sensitivity of 85% and specificity of 56% [14]. Novel biomarkers, such as copeptin, progranulin, and MxA1 protein are also gaining interest in distinguishing between bacterial and viral CAP [12, 34–36]. A rapid test, combining MxA1 and CRP, showed promising diagnostic accuracy in several studies [37–39]. However, despite impressive research results, the question of their availability and affordability for routine clinical practice remains.

Another notable finding from our study was a higher likelihood of vasopressor prescription within 24 h of admission in cases of bacterial CAP. While this risk factor has not been described by other researchers, Serrano Fernández L. et al. revealed a higher ICU admission rate and severity score according to PORT and CURB‐65 among hospitalized patients with bacterial CAP compared with COVID‐19 [9].

The search for rapid and affordable tools for the differential diagnosis of bacterial and viral CAP is particularly relevant, especially in light of the observed overprescription of antibiotics during the COVID‐19 pandemic [5, 20, 21]. Bacterial co‐infections are not typically characteristic of COVID‐19 [40, 41]. Therefore, the ability to reliably distinguish between bacterial and viral CAP, including COVID‐19, before etiological verification, offers the opportunity for early initiation of adequate therapy. This can help reduce the unjustified antibiotics use and mitigate the selection of antibiotic resistance.

The developed model consists of simple and widely accessible parameters. Therefore, its application is feasible in hospitals with varying levels of equipment. Its use is particularly relevant during seasonal peaks in viral infections, when limiting unnecessary antibiotic prescriptions is especially important. However, it should be noted that the use of such a model should not limit or replace the use of available etiologic tests. In addition to high‐quality culture tests, rapid tests should be widely implemented, including urinary antigen tests for *S. pneumoniae* and *L. pneumophila*. Modern molecular tests, including rapid PCR panels, are likely to improve pathogen‐specific treatment [42].

However, the study has several limitations, primarily its retrospective nature. The viral pneumonia group predominantly consisted of patients with COVID‐19, which may make it difficult to extrapolate the data to patients with other viral pathogens. The low number of patients with viral CAP of non‐COVID etiologies reflects the limited availability of PCR testing for respiratory viruses in our country during the pre‐COVID era. There were also gaps in the collected data, necessitating methods to handle missing data, including replacing them with median values. Serum albumin was not included into analysis due to inconsistent availability and limited measurements across centers; total protein was used instead, although albumin may be a more clinically informative marker. Furthermore, recruiting comparable groups of patients in terms of disease severity proved challenging, as there is currently no universal severity scale validated for bacterial and viral CAP, including COVID‐19. Inclusion of patients only with confirmed etiology was implied by the design of our study; however, this decision likely resulted in the selection of more severe patients into the bacterial CAP group, since the probability of detecting the pathogen in such patients is higher than in milder cases. Additionally, because of the small number of patients with mixed infection, they were analyzed in bacterial CAP group. The clinical reasoning for this decision was the need for identification of patients requiring antibiotic administration, although a tool, capable of assessing mixed infection, might be more precise.

## 5. Conclusions

Distinguishing between CAP of different etiologies in adults poses a challenge, especially with the increasing incidence of viral pathogens. The developed models in our study incorporated parameters that are readily available in routine clinical practice. These models can be implemented in various hospital units for rapid triage of admitted patients and decision‐making regarding antibiotic prescription before the etiology of CAP has been established. However, despite demonstrating good diagnostic accuracy, the model requires further validation in a larger sample size and in a prospective cohort of patients. Additionally, determining the added value of new markers, if available, also remains an area of further interest for research in this field.

## Author Contributions

Svetlana Rachina: conceptualization, funding acquisition, investigation, methodology, writing – original draft, writing – review and editing.

Daria Strelkova: conceptualization, funding acquisition, investigation, methodology, project administration, writing – original draft, writing – review and editing.

Anastasia Yasneva: data curation, investigation, writing – review and editing.

Olga Kupriushina: data curation, investigation, writing – review and editing.

Sergey Avdeev: conceptualization, funding acquisition, methodology writing – review and editing.

Anna Vlasenko: formal analysis, visualization, writing – review and editing.

Lyudmila Fedina: data curation, investigation, writing – review and editing.

Olga Ivanova: data curation, investigation, writing – review and editing.

Ivan Zakharenkov: data curation, investigation, writing – review and editing.

Inna Kaledina: data curation, investigation, writing – review and editing.

Nataliia Ananicheva: project administration, supervision, writing – review and editing.

## Funding

This study was supported by Russian Science Foundation, 10.13039/501100006769, 23‐25‐00422.

## Conflicts of Interest

The authors declare no conflicts of interest.

## Supporting information


**Supporting Information** Additional supporting information can be found online in the Supporting Information section. Regression equations Z1‐4. Table S1. Description of patients’ cohorts. Table S2. Etiology of CAP in adults. Figure S1. Nomogram of predicting bacterial vs viral CAP based on objective variables (dataset with removed missing data). Figure S2. Nomogram of predicting bacterial vs. viral CAP based on objective variables (dataset with replacement of missing data with the median values). Figure S3. Nomogram of predicting bacterial vs. viral CAP based on objective and subjective variables (dataset with removed missing data). Figure S4. Nomogram of predicting bacterial vs viral CAP based on objective and subjective variables (dataset with replacement of missing data with the median values).

## Data Availability

The data that support the findings of this study are available from the corresponding author upon reasonable request.
