# Supplementary material for: Distinguishing Between Bacterial and Viral Community‐Acquired Pneumonia in Hospitalized Adults: The Importance of Clinical, Laboratory, and Imaging Findings
Source: Pulm Med. 2026 Jun 21;2026:8552717. doi: 10.1155/pm/8552717 (PMC13284490; doi:10.1155/pm/8552717)
Supplement: Supplementary file 1 — Supporting Information Additional supporting information can be found online in the Supporting Information section. Regression equations Z1‐4. Table S1. Description of patients’ cohorts. Table S2. Etiology of CAP in adults. Figure S1. Nomogram of predicting bacterial vs viral CAP based on objective variables (dataset with removed missing data). Figure S2. Nomogram of predicting bacterial vs. viral CAP based on objective variables (dataset with replacement of missing data with the median values). Figure S3. Nomogram of predicting bacterial vs. viral CAP based on objective and subjective variables (dataset with removed missing data). Figure S4. Nomogram of predicting bacterial vs viral CAP based on objective and subjective variables (dataset with replacement of missing data with the median values). [file PM-2026-8552717-s001.docx]

**Supplement materials**

Regression equations

Objective variables, with missing data removed

Z1 = -0.09 * TP + 4.12 * UI + 1.57 * DC + 0.05 * HR + 3.9 * V + 0.05 * N/L, (1)

Objective variables, with missing data replaced by median values

Z2 = -0.10 * TP + 3.31 * UI + 1.28 * DC + 0.04 * HR + 2.5 * V + 0.05 * N/L + 0.04 * SU -1.1 * P, (2)

Combination of objective and subjective variables, with missing data removed

Z3 = -0.06 * TP + 4.13 * UI + 1.20 * DC + 0.04 * HR + 3.6 * V + 0.05 * N/L -1.91 * GW + 2.56 * C, (3)

Combination of objective and subjective variables, with missing data replaced by median values

Z4 = -0.07 * TP + 3.01 * UI + 1.15 * DC + 0.03 * HR + 2.1 * V + 0.05 * N/L -1.58 * GW + 3.02 * C, (4)

Note: TP – Total serum protein, UI – Unilateral infiltration on chest X-ray/CT, DC – Decreased consciousness, HR – Heart rate, V – Vasopressors use in 24 hours of admission, N/L – Neutrophils/lymphocytes ratio, SU – Serum urea level, P – Proteinuria, GW - General weakness, C - Chills

Table 1S. Description of patients’ cohorts

|  | Bacterial CAP | | Viral CAP | | *Р* | *SMD* |
| --- | --- | --- | --- | --- | --- | --- |
|  | n* | Value | n* | Value |  |  |
| **Demographic characteristics, place of admission, complaints upon admission** | | | | | |  |
| Age, years | 100 | 51.0 (18.9) | 300 | 51.5 (18.4) | - | -0.07 |
| Male | 100 | 53/100 (53%) | 300 | 143/300 (47.7%) | - | 0.09 |
| ICU/general ward | 100 | 66/100 (66%) | 300 | 201/300 (67%) | - | 0.02 |
| *Dyspnea* | *100* | *71/100 (71%)* | *300* | *168/300 (56%)* | *0.01* | *0.30* |
| Cough | 100 | 63/100 (63%) | 300 | 213/300 (71%) | 0.17 | -0.16 |
| *Presence of sputum* | *100* | *49/100 (49%)* | *300* | *63/300 (21%)* | *<0.001* | *0.62* |
| *Chest pain* | *100* | *41/100 (41%)* | *300* | *18/300 (6%)* | *<0.001* | *0.91* |
| Increased temperature | 100 | 62/100 (62%) | 300 | 213/300 (71%) | 0.10 | -0.19 |
| *Anosmia* | *100* | *0* | *300* | *18/300 (6%)* | *0.009* | *-0.28* |
| *Rhinitis* | *100* | *14/100 (14%)* | *300* | *6/300 (2%)* | *<0.001* | *0.45* |
| *Chills* | *100* | *38/100 (38%)* | *300* | *6/300 (2%)* | *<0.001* | *1.00* |
| *General weakness* | *100* | *13/100 (13%)* | *300* | *195/300 (65%)* | *<0.001* | *-1.25* |
| *Decreased level of consciousness* | *100* | *22/100 (22%)* | *300* | *15/300 (5%)* | *<0.001* | *0.51* |
| **Comorbidities** | | | | | |  |
| Charlson comorbidity index, points | 100 | 2.0 (0.0; 4.0) | 300 | 2.0 (0.0; 4.0) | - | 0.03 |
| *Arterial hypertension* | *100* | *27/100 (27%)* | *300* | *186/300 (62%)* | *<0.001* | *-0.74* |
| Myocardial infarction | 100 | 9/100 (9%) | 300 | 33/300 (11%) | 0.51 | -0.07 |
| *Congestive heart failure* | *100* | *18/100 (18%)* | *300* | *27/300 (9%)* | *0.03* | *0.25* |
| Stroke or transient ischemic attack | 100 | 11/100 (11%) | 300 | 39/300 (13%) | 0.56 | -0.06 |
| *Chronic obstructive pulmonary disease* | *100* | *16/100 (16%)* | *300* | *15/300 (5%)* | *<0.001* | *0.37* |
| *Chronic hepatitis* | *100* | *15/100 (15%)* | *300* | *6/300 (2%)* | *<0.001* | *0.47* |
| Diabetes mellitus | 100 | 13/100 (13%) | 300 | 51/300 (17%) | 0.30 | -0.12 |
| Chronic kidney disease, stage С3а-С5 | 100 | 9/100 (9%) | 300 | 36/300 (12%) | 0.42 | -0.09 |
| **Physical examination upon admission** | | | | | |  |
| Temperature, С° | 94 | 37.0 (36.6; 37.8) | 282 | 36.8 (36.6; 37.5) | 0.05 | 0.24 |
| SpO_2_, % | 86 | 92.0 (87.2; 96.8) | 293 | 94.0 (90.0; 96.0) | 0.002 | -0.34 |
| *Systolic blood pressure, mm Hg* | *100* | *115 (100; 130)* | *300* | *120 (112; 130)* | *<0.001* | *-0.40* |
| *Diastolic blood pressure, mm Hg* | *100* | *70 (60; 80)* | *300* | *76 (70; 82)* | *<0.001* | *-0.52* |
| *Heart rate, per min* | *99* | *100 (85; 115)* | *300* | *87 (80; 98)* | *<0.001* | *0.70* |
| *Respiratory rate, per min* | *98* | *22.0 (18.2; 26.0)* | *298* | *20.0 (20.0; 22.0)* | *<0.001* | *0.50* |
| *Coarse crackles* | *100* | *44/100 (44%)* | *210* | *21/300 (7%)* | *<0.001* | *0.95* |
| *Wheezes* | *100* | *29/100 (29%)* | *210* | *21/300 (7%)* | *<0.001* | *0.59* |
| *Fine crackles* | *100* | *24/100 (24%)* | *210* | *3/300 (1%)* | *<0.001* | *0.72* |
| *Decreased breath sound* | *100* | *35/100 (35%)* | *210* | *36/300 (12%)* | *<0.001* | *0.55* |
| **Instrumental findings upon admission** | | | | | |  |
| *Chest X-ray was performed* | *100* | *69/100 (69%)* | *300* | *57/300 (19%)* | *<0.001* | *1.17* |
| *Chest CT was performed* | *100* | *53/100 (53%)* | *300* | *294/300 (98%)* | *<0.001* | *-1.22* |
| *Chest X-ray/CT: bilateral infiltration* | *100* | *51/100 (51%)* | *300* | *267/300 (89%)* | *<0.001* | *-0.92* |
| *Chest X-ray/CT: infiltration localisation not specified* | *100* | *0* | *300* | *18/300 (6%)* |  | *-0.37* |
| *Chest X-ray/CT: left-sided infiltration* | *100* | *19/100 (19%)* | *300* | *9/300 (3%)* |  | *0.52* |
| *Chest X-ray/CT: right-sided infiltration* | *100* | *30/100 (30%)* | *300* | *3/300 (1%)* |  | *0.85* |
| *Chest X-ray/CT: pleural effusion* | *100* | *26/100 (26%)* | *300* | *30/300 (10%)* | *<0.001* | *0.42* |
| *Chest CT: ground-glass opacities* | *53* | *18/53 (34%)* | *294* | *279/294 (95%)* | *<0.001* | *-1.67* |
| *Chest CT: consolidations* | *53* | *38/53 (72%)* | *294* | *132/294 (45%)* | *0.001* | *0.56* |
| *Heart rate on ECG* | *64* | *100 (80.0; 116.2)* | *149* | *88 (72.0; 99.0)* | *<0.001* | *0.70* |
| **Laboratory findings upon admission** | | | | | |  |
| *Red blood cells, 10^12/L* | *100* | *4.2 (3.5; 4.5)* | *299* | *4.5 (4.0; 4.9)* | *<0.001* | *-0.18* |
| *Hemoglobin, g/L* | *72* | *130 (118; 141)* | *298* | *137 (123; 150)* | *0.017* | *-0.29* |
| *Haematocrit, %* | *99* | *36.8 (32.2; 40.8)* | *297* | *39.7 (35.9; 43.2)* | *<0.001* | *-0.47* |
| MCV, fl | 61 | 89.0 (86.0; 94.3) | 292 | 88.2 (84.8; 93.0) | 0.180 | 0.21 |
| MCH, pg | 60 | 30.8 (29.1; 32.6) | 291 | 30.6 (29.0; 32.1) | 0.790 | -0.05 |
| *Blood leukocytes, 10^9/L* | *100* | *10.0 (5.6; 15.1)* | *300* | *6.2 (4.7; 9.0)* | *<0.001* | *0.52* |
| *Blood neutrophils, 10^9/L* | *69* | *7.8 (4.8; 12.8)* | *278* | *4.3 (3.1; 7.1)* | *0.002* | *0.50* |
| *Blood neutrophils/lymphocytes ratio* | *69* | *6.6 [3.5;14.8]* | *277* | *3.9 [2.4;7.7]* | *<0.001* | *0.58* |
| *Blood lymphocytes, 10^9/L* | *71* | *0.9 (0.5; 1.5)* | *296* | *1.1 (0.7; 1.6)* | *0.227* | *-0.12* |
| Platelets, 10^9/L | 100 | 199 (126; 245) | 292 | 172 (133; 221) | 0.09 | 0.18 |
| *CRP, mg/L* | *66* | *129.9 (33.9; 282.4)* | *297* | *78.4 (35.4; 147.3)* | *<0.001* | *0.59* |
| *Procalcitonin >0.5 ng/mL* | *47* | *35/47 (74%)* | *212* | *36/212 (17%)* | *<0.001* | *1.41* |
| Alanine transaminase, U/L | 92 | 35.4 (19.9; 60.3) | 293 | 39.8 (23.5; 63.0) | 0.78 | -0.03 |
| Aspartate transaminase, U/L | 93 | 45.0 (25.0; 98.0) | 296 | 47.0 (31.0; 74.4) | 0.36 | 0.14 |
| Serum glucose, mmol/L | 93 | 6.6 (5.2; 8.3) | 286 | 6.7 (5.7; 9.0) | 0.16 | -0.18 |
| *Total serum protein, g/L* | *79* | *63.0 (56.5; 68.0)* | *272* | *69.8 (63.7; 74.5)* | *<0.001* | *-0.69* |
| Sodium, mmol/L | 74 | 136.0 (133.2; 141.0) | 181 | 137 (132; 140) | 0.725 | 0.047 |
| *Serum creatinine, μmol/L* | *99* | *121.0 (85.1; 193.4)* | *295* | *91.7 (72.8; 124.8)* | *<0.001* | *0.44* |
| *Serum urea, mmol/L* | *95* | *9.6 (6.1; 14.8)* | *292* | *5.6 (3.9; 9.0)* | *<0.001* | *0.51* |
| *Total bilirubin, μmol/L* | *54* | *15.1 (9.0; 22.0)* | *287* | *10.1 (6.4; 14.0)* | *0.15* | *0.27* |
| *Direct bilirubin, μmol/L* | *27* | *7.6 (3.4; 12.6)* | *137* | *3.9 (2.5; 5.7)* | *0.62* | *0.22* |
| *International normalized ration* | *48* | *1.2 (1.1; 1.4)* | *286* | *1.0 (0.9; 1.2)* | *0.002* | *0.53* |
| *D-dimer, ng/mL* | *17* | *1714 (430; 6243)* | *261* | *549 (293; 1179)* | *<0.001* | *0.71* |
| *рН, arterial blood* | *50* | *7.4 (7.3; 7.4)* | *112* | *7.4 (7.4; 7.5)* | *0.001* | *-0.58* |
| *Proteinuria* | *100* | *31/100 (31%)* | *300* | *147/300 (49%)* | *0.002* | *-0.35* |
| *Leukocyturia* | *100* | *9/100 (9%)* | *300* | *54/300 (18%)* | *0.037* | *-0.26* |
| *Ketonuria* | *100* | *10/100 (10%)* | *300* | *69/300 (23%)* | *0.033* | *-0.26* |
| **Treatment upon admission** | | | | | |  |
| *Mechanical ventilation* | *100* | *22/100 (22%)* | *300* | *21/300 (7%)* | *<0.001* | *0.44* |
| *Vasopressors in 24 hours of admission* | *100* | *13/100 (13%)* | *300* | *3/300 (1%)* | *<0.001* | *0.46* |
| Infusion therapy | 100 | 64/100 (64%) | 300 | 195/300 (65%) | 0.95 | -0.02 |
|  |  |  |  |  |  |  |
| CURB 65 | 93 | 2.0 [1.0;2.0] | 290 | 1.0 [0;2.0] | *<0.001* | *0.16* |
| Length of stay | 100 | 12.0 [6.0;17.2] | 300 | 9.0 [6.0;14.0] | 0.90 | 0.02 |

*Note:* *number of patients in whom this parameter was determined

Table 2S. Aetiology of CAP in adults

| Pathogen | n (%) |
| --- | --- |
| Bacterial CAP group | |
| *S. pneumoniae*, n (%) | 42/100 (42%) |
| *K. pneumoniae*, n (%) | 10/100 (10%) |
| *S. aureus*, n (%) | 7/100 (7%) |
| *L. pneumophila*, n (%) | 5/100 (5%) |
| *M. pneumoniae*, n (%) | 4/100 (4%) |
| *P. aeruginosa*, n (%) | 3/100 (3%) |
| *E. coli*, n (%) | 2/100 (2%) |
| *C. pneumoniae*, n (%) | 2/100 (2%) |
| *H. influenzae*, n (%) | 1/100 (1%) |
| *Acinetobacter* spp., n (%) | 1/100 (1%) |
| *S. pneumoniae* + other bacterial pathogens^1^, n (%) | 15/100 (15%) |
| Mixed bacterial aetiology, except *S. pneumoniae,* n (%) | 8/100 (8%) |
| Viral CAP group | |
| SARS-CoV-2*,* n (%) | 290/300 (96.6%) |
| rhinovirus*,* n (%) | 3/300 (1%) |
| metapneumovirus*,* n (%) | 2/300 (0.7%) |
| influenza A virus*,* n (%) | 2/300 (0.7%) |
| human respiratory syncytial virus (RSV) *,* n (%) | 1/300 (0.3%) |
| other human coronaviruses*,* n (%) | 1/300 (0.3%) |
| influenza B virus + RSV*,* n (%) | 1/300 (0.3%) |

^1^ *S. pneumoniae + S. aureus – 4 cases, S. pneumoniae + K. pneumoniae – 2 cases, S. pneumoniae + Acinetobacter baumannii – 2 cases, S. pneumoniae + E. coli – 1 case, S. pneumoniae + Haemophilus influenzae – 1 case, S. pneumoniae + Haemophilus parainfluenzae – 1 case, S. pneumoniae + Neisseria meningitidis – 1 case, S. pneumoniae + K. pneumoniae + Haemophilus influenzae – 1 case, S. pneumoniae + S. aureus + H. influenzae + K. pneumoniae – 1 case, S. pneumoniae + S. aureus + K. pneumoniae – 1 case*; in 9 cases coinfection with viruses, mainly rhinovirus was revealed





Figure 1S. Nomogram of predicting bacterial vs viral CAP based on objective variables (dataset with removed missing data)





Figure 2S. Nomogram of predicting bacterial vs viral CAP based on objective variables (dataset with replacement of missing data with the median values)





Figure 3S. Nomogram of predicting bacterial vs viral CAP based on objective and subjective variables (dataset with removed missing data)





Figure 4S. Nomogram of predicting bacterial vs viral CAP based on objective and subjective variables (dataset with replacement of missing data with the median values)
